# Persistent Activation of Innate Immunity in Patients with Primary Antibody Deficiencies

**DOI:** 10.1155/2020/8317671

**Published:** 2020-11-20

**Authors:** Gerasimina Tsinti, Demosthenes Makris, Anastasios E. Germenis, Matthaios Speletas

**Affiliations:** ^1^Department of Immunology & Histocompatibility, Faculty of Medicine, School of Health Sciences, University of Thessaly, Larissa, Greece; ^2^Department of Critical Care Medicine, University Hospital of Larissa, Faculty of Medicine, School of Health Sciences, University of Thessaly, Larissa, Greece

## Abstract

Primary antibody deficiencies (PAD) represent a heterogeneous group of disorders, with common variable immunodeficiency being the most common with clinical significance. The main phenotypic defect resides in the inability of B cells to produce antibodies, and the cornerstone of therapy is immunoglobulin replacement treatment in order to fight infections. However, the management of the other inflammatory manifestations is inadequate, reinforcing the hypothesis that a complex genetic background affecting additional cell populations, such as polymorphonuclear cells (PMN) and monocytes, influences the expression of the clinical phenotype of the disease. In this study, we investigated by flow cytometry in different conditions (resting state, and after isolation and incubation, with and without stimuli) the expression pattern of several markers on PMN and monocytes, indicative of their maturation, capacity for chemotaxis, adhesion, opsonization, migration, and phagocytosis in 25 PAD patients, 12 healthy blood donors, and 4 septic patients. In this context, we also analyzed patients before and after the initiation of replacement treatment, as well as an untreated patient in different clinical conditions. Interestingly, we observed that PAD patients exhibit a chronic activation status of the innate immunity compartment, along with several differences in the expression of activation, maturation, and adhesion markers, with respect to different clinical conditions. Moreover, immunoglobulin replacement treatment had a favorable effect on PMN, as it was expressed by a more mature and less activated phenotype on basal state cells, and an enhanced activation capacity after LPS exposure. Thus, we conclude that PAD patients display a persistent innate immune cell activation, which is probably associated with the chronic inflammatory stress, usually observed in these disorders.

## 1. Introduction

Primary antibody deficiencies (PAD) are a heterogeneous group of disorders where the common characteristic and main phenotypic defect resides in the inability of B cells to differentiate and produce antibodies. The most common and clinically important PAD is common variable immunodeficiency (CVID), which is sporadic with unknown genetic etiology in the majority (approximately 80%) of cases [1–3]. Affected patients suffer from frequent and recurrent infections, while they also display a high prevalence of autoimmune manifestations, granulomas formation, benign lymphoproliferation, and malignancies, especially lymphomas [1, 2]. Immunoglobulin replacement therapy is the cornerstone for the management of PAD patients, resulting in a substantial reduction of the frequency and the severity of infections, but it has a limited effect on the manipulation of the other inflammatory manifestations of the disease [1, 3].

Recent studies support the notion that the innate immunity might contribute to disease pathogenesis, since altered phenotype and function of monocytes [4], dendritic cells (DCs) [5], and natural killer cells [6] have been reported in CVID patients. However, the contribution of polymorphonuclear cells (PMN) in CVID remains rather obscure, since until now only a few studies have analyzed PMN immunophenotyping and function in disease pathogenesis and/or phenotype, with contradictory results [7–11].

PMN are the major effector cells in host defense against invading microorganisms, contributing also to the activation and regulation of B cell homeostasis and differentiation, through the production of appropriate cytokines such as BAFF and APRIL [12]. PMN defects result in the emergence of severe and well-characterized immunodeficiencies, such as leukocyte adhesion deficiencies (LAD), chronic granulomatous disease (CGC), and Chediak-Higashi syndrome (reviewed by Dinauer) [13]. On the other hand, PMN during severe inflammatory conditions, such as sepsis, exhibit disturbed and impaired functions leading to SIRS, immunoparalysis, and eventually to the deterioration of patients' clinical state [14]. Thus, it is a challenge to explore the possible contribution of PMN to PAD pathogenesis and/or phenotype, since such an understanding might substantially affect the improved knowledge of the disease and patients' management.

However, there are many challenges to be faced when studying the innate immunity in PAD patients, especially those suffering from CVID. The first and most important is the variable nature of the disease that is reflected in a diverse clinical phenotype of the affected patients [1, 15]. This varied phenotype may be the cause for the different, and even contradicting, results of several studies, leading to divergent conclusions. The second challenge is the rare nature of the disease that affects the power of the statistical comparisons. Moreover, CVID patients usually require immediate immunoglobulin replacement therapy, and, as a consequence, the number of newly diagnosed patients not receiving therapy is even smaller. As a result, the great majority of the published studies refer to patients under immunoglobulin replacement treatment [9–11, 16]. The third challenge, but not less important, is the effect of experimental manipulation of PMN, since they are short-lived cells in vivo and extremely sensitive to phenotypic changes in vitro, as described [17–19].

Bearing in mind the contradictory results in the literature, the aim of our study was to further elucidate whether the innate immune cells, especially PMN and monocytes, are affected and/or contribute to the clinical phenotype of PAD patients. For this purpose, we evaluated by flow cytometry the expression pattern of specific markers on PMN and monocytes, indicative of their maturation stage, as well as of their capacity for chemotaxis, adhesion, opsonization, migration, and phagocytosis in different conditions (resting state, and after isolation and incubation, with and without stimuli), in order to assess whether the activation of these cells is associated with the chronic inflammatory stress that is rather a typical characteristic of these disorders.

## 2. Materials and Methods

### 2.1. Subjects

Twenty-five PAD patients (male/female: 9/16, mean age: 41.3 years, range: 14-66) were enrolled in the study (Table S1). Among them, 23 patients fulfilled the diagnostic criteria of CVID [20], while two patients were eventually recognized as suffering from CTLA4-depedent immune dysregulation syndrome, carrying specific mutations, as described by us recently [21]. Six patients (24.0%, all with CVID, group A) were analyzed at diagnosis (male/female: 2/4, mean age: 44.5 years, range: 14-66), while the remaining 19 patients (76.0%, male/female: 7/12, mean age: 40.3 years, range: 19-65) were receiving either intravenous (IVIG, 3 patients) or facilitated subcutaneous immunoglobulin (fSCIG, 16 patients) replacement treatment (group B). Nine patients (36.0%, 2 newly diagnosed) suffered from chronic respiratory disease (CRD, obstructive and/or restrictive disease) receiving inhaled bronchodilators, and 12 patients (48.0%, 3 newly diagnosed) had bronchiectasis, with the majority of them—10 out of 12 (83.3%)—displaying CRD. Six patients (24.0%, 2 newly diagnosed) had enteropathy, 5 patients (20.0%, all under replacement treatment) exhibited granulomatous disease, one patient under IVIG (4.0%) had complications from nodular regenerative hyperplasia (NRH) of the liver, and 2 (8.0%) had a medical history of neoplasia, including a patient with lymphoma and a newly diagnosed patient with colon adenocarcinoma. Twelve patients (48.0%, 1 newly diagnosed) displayed benign lymphoproliferation (splenomegaly and/or lymphadenopathy), while three more had been subjected to splenectomy in the past (two for diagnostic purposes, i.e., a massive splenomegaly with hypersplenism and a differential diagnosis of lymphoma, and one more for the management of resistant autoimmune thrombocytopenic purpura (ATP)). Additionally, 11 patients (44.0%, 2 newly diagnosed) experienced one or multiple autoimmune manifestations, including nine (36.0%) with thyroid disease, three (12.0%) with ATP, three (12.0%) with pernicious anemia, one with psoriasis (complicated also by psoriatic arthritis), one with myelitis, one with arthritis, one with vitiligo, and one with recurrent autoimmune hemolytic anemia.

At the time of analysis, one patient was receiving immunosuppressive treatment due to resistant/recurrent ATP (2 g mycophenolate mofetil per day) and another one was receiving a low dose of prednisolone (10 mg per day) due to an exacerbation of restrictive CRD. Three CVID patients carried the *TNRSF13B/TACI*-p.C104R mutation in heterozygous state [22], another one patient carried the *IKZF1-*p.His191Tyr mutation also in heterozygous state, while four out of six newly diagnosed CVID patients were also analyzed at least five months (mean: 6 months, range: 5-7) after the initiation of replacement treatment.

The results of the analysis of PAD patients were compared to a cohort of 12 age- and sex-matched (male/female: 4/8, mean age: 40.8 years, range: 23-67) healthy individuals, while 4 septic patients from an intensive care unit (ICU) (male/female: 3/1, mean age: 65.5 years, range: 61-72), two without and two under corticosteroid medication at the time of blood collection, served as a disease control group.

The study was conducted in accordance with the principles of the Helsinki declaration and was approved by the Institutional Review Board of the University Hospital of Larissa. Written informed consent was obtained from each individual or an accompanying relative, as in patients where consent was not legally applicable; the procedures followed were in accordance with institutional guidelines.

### 2.2. Culture Assays and Immunophenotyping

Seven to eight milliliters (mL) of heparinized whole-blood samples were separated in three parts. The first part (2 mL) was immediately used for immunophenotyping of innate immune cells (polymorphonuclear cells/PMN and monocytes) at resting (basal) state. The second part (4 mL) was used for PMN isolation by 1077/1119 Histopaque double-gradient density centrifugation (Histopaque; Sigma-Aldrich, St. Louis, Missouri, USA) at room temperature (RT), and the immunophenotyping was performed on the cells of the lower interface (normal density PMN). The third part (0.5-1 mL) was used in culture assays in order to evaluate the response of PMN to stimuli; in particular, 2 × 10^5^ whole blood cells, including at least 1 − 1.5 × 10^5^ PMN, were seeded on a 24-well tissue culture plate, in a total volume of 500 microliters (*μ*L) Iscove's Basal Medium (Biochrom, Berlin, Germany), containing 6% heat-inactivated normal human serum, with and without lipopolysaccharide (LPS) (100 ng/ml, *E. Coli* serotype 026:B6, Sigma- Aldrich), and were incubated in a humidified atmosphere (37°C, 8% CO_2_) for one hour.

Immunophenotyping was performed by flow cytometry on Coulter FC-500 instrument (Epics XL-MCL, 4 color analysis, Beckman-Coulter/BC, Hialeah, FL, USA) using a multistaining protocol and commercially available reagents. Mouse anti-human immunoglobulin G (IgG) and M (IgM) monoclonal antibodies were used to detect molecules that reacted with CD10 (clone: ALB1), CD11b (clone: Bear1), CD11c (clone: BU15), CD14 (clone: RMO52), CD16 (clone: 3G8), CD18 (clone: 7E4), CD64 (clone: 22), and CD66b (clone: 80H3). All of the above antibodies were purchased by BC and were conjugated with the appropriate fluorochrome (fluorescein isothiocyanate, FITC; phycoerythrin, PE; PE-cyanine5 PE-Cy5). Erythrocytes were lysed using NH_4_Cl/KHCO_3_ lysis solution, and the cells were washed twice with PBS and stained with the aforementioned monoclonal antibodies for 15 minutes. The percentage of fluorescent cells and the mean fluorescence intensity (MFI) were determined in each case corrected for background fluorescence, using FITC, PE, and PE-Cy5-labelled control antibodies. The data analyzed were the percentile expression (%) of each marker and the intensity of expression determined by the equation: (%)expression × MFI/100.

The expression markers on monocytes were evaluated only in resting state. On the other hand, we assessed the PMN immunophenotyping in four different conditions: (1) resting (basal) state, where PMN had not undergone any treatment; (2) normal density PMN, whose phenotype reflects changes due to the isolation procedure; (3) culture-control PMN, whose phenotype reflects changes under culture conditions without any trigger; and (4) culture-LPS PMN, whose phenotype reflects changes under LPS trigger.

### 2.3. Statistical Analysis

The Wilcoxon-matched paired analysis, non-parametric Mann–Whitney test, and Spearman correlation analyses were performed for the comparison of different study groups. Statistical analyses and graphs were made on the GraphPad-6 Prism software (version 6, CA, USA). For all analyses, alpha was set at 0.05 (2-sided).

## 3. Results

### 3.1. Activation of Innate Immune Cells in PAD Patients

**Figure 1:**
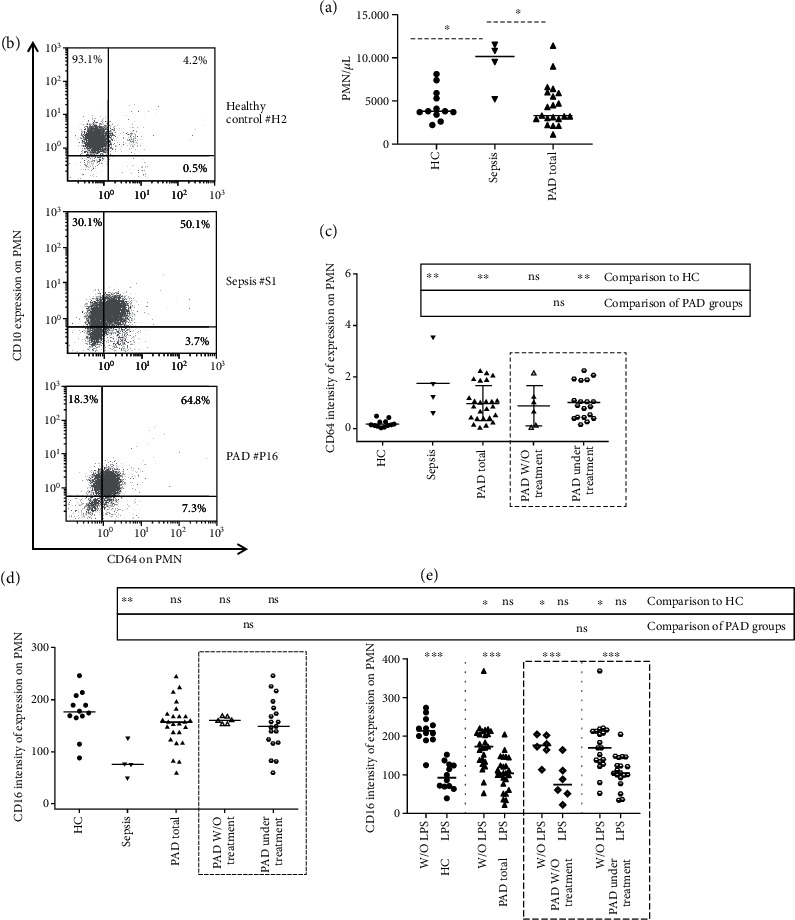
(a) PMN absolute numbers (per *μ*L) of whole peripheral blood of the patients and controls of the study. (b) Representative dot plots of CD64 expression on PMN. (c) CD64 intensity of expression on polymorphonuclear cells (PMN) of the patients and controls of the study. (d) CD16 intensity of expression on whole-blood PMN of the patients and controls of the study at basal state. (e) After incubation of whole blood without and with LPS. The lines represent the median values. Statistical analyses were performed by the Mann–Whitney *U* test. The Wilcoxon-matched paired analysis was performed to compare same subjects under culture conditions; ∗*p* < 0.05; ∗∗*p* < 0.01; ns, not significant.

**Figure 2:**
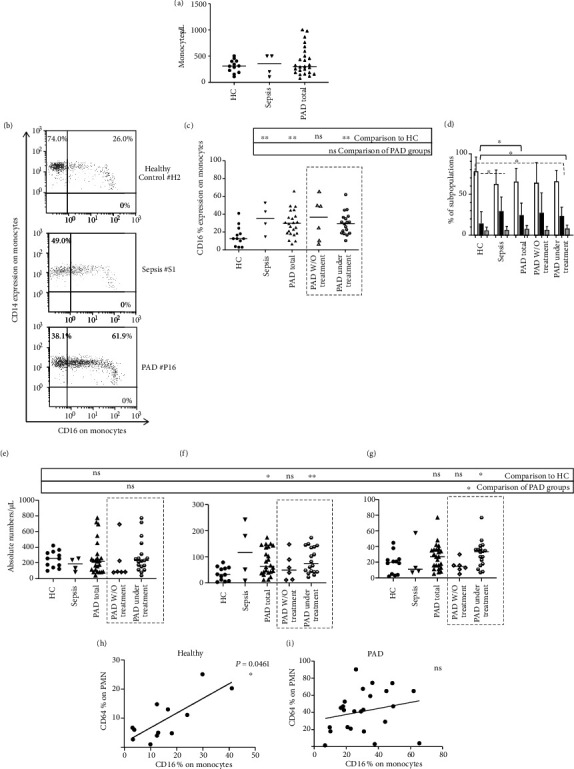
(a) Monocyte absolute numbers (per *μ*L) of whole peripheral blood of the patients and controls of the study. (b) Representative dot plots of CD16 expression on whole-blood monocytes at basal state. (c) CD16 percentile expression on monocytes of the patients and controls of the study. (d) Percentages of classical, intermediate, and non-classical subpopulations within the monocyte population in whole-blood basal state condition, for the patients and controls of the study: White columns represent classical monocytes, black columns the intermediate monocytes, and grey columns the non-classical monocytes. (e) Absolute numbers of classical, (f) intermediate, (g) and non-classical monocytes. (h) Correlation of CD64 percentage of expression on PMN to CD16 percentage of expression on monocytes on healthy controls and (i) in PAD patients. Statistical analyses were performed by the Mann–Whitney *U* test and Spearman correlation analysis; ∗*p* < 0.05; ∗∗*p* < 0.01; ns, not significant.

The most important finding of our study was the demonstration of a steady activation status of both PMN and monocytes in patients with PAD, irrespective of the patients' treatment status. In particular, PAD patients displayed at the basal state a higher CD64 expression on PMN and a higher CD16 expression on monocytes, compared to healthy controls, despite the absence of an acute infection, as it was confirmed by CRP levels that were into normal range for the majority of patients (with an exception of a slight increase in some patients with CRD). Moreover, as presented in Figures 1, and 2, the pattern of expression of both molecules in PAD patients was rather similar with those observed in sepsis ones.

As demonstrated in previous studies, including ours, CD64 expression on PMN is generally stable even after isolation or incubation conditions [18, 19]. Thus, as expected, CD64 levels on PMN of PAD patients differed significantly compared to healthy controls also in the other experimental conditions, i.e., isolation and incubation with and without LPS (Figure S1).

CD16 is expressed on both resting PMN and activated monocytes, while its downregulation on PMN is indicative of their activation [23, 24]. Considering CD16 expression on PMN in our cohort, it is worth noting that septic patients expressed significantly lower levels of CD16 at basal state (indicative also of their activation), while PAD patients displayed a significant downregulation of CD16 only on PMN after incubation, without LPS (Figure 1). Although the same pattern of CD16 expression on PMN in basal state was also observed, the difference compared to healthy controls did not reach levels to be significant (Figure 1). As mentioned above, our PAD patients displayed a significant increase in CD16 expression on monocytes (percentile and absolute numbers), indicative of their steady activation status (Figure 2, Table S2). Further analysis of monocytes subpopulations with respect to CD14 and CD16 co-expression patterns revealed that PAD patients under replacement treatment exhibited higher percentages and absolute numbers of intermediate (CD14^++^/CD16^+^) and non-classical (CD14^+^/CD16^+^) monocytes compared to healthy controls. On the other hand, despite differences in the percentages, no significant differences on the absolute numbers of classical (CD14^++^/CD16^−^) monocytes in PAD patients compared to healthy controls were observed (Figure 2 and Table S2).

Considering the other analyzed molecules, no significant differences were observed between PAD patients and controls (Tables S3-S6). However, sepsis patients displayed a significant increase of CD66b and a downregulation of CD10 and CD16 compared to HC (Tables S3-S6), similar to previous studies [25, 26].

We would like to note that the isolated PMN being analyzed in our study refer to normal density and not to low density ones. The later PMN populations have been recognized after density gradient centrifugation in the PBMC fraction, and they have been analyzed in some pathological conditions [27, 28]. However, in our cohort, the frequency of such populations was extremely low (0.2-0.5%), namely in the limits of an artifact, and we considered that such populations were not adequate for further statistical analyses.

### 3.2. Effect of Immunoglobulin Replacement Treatment on the Phenotype of PMN and Monocytes in PAD Patients

**Figure 3:**
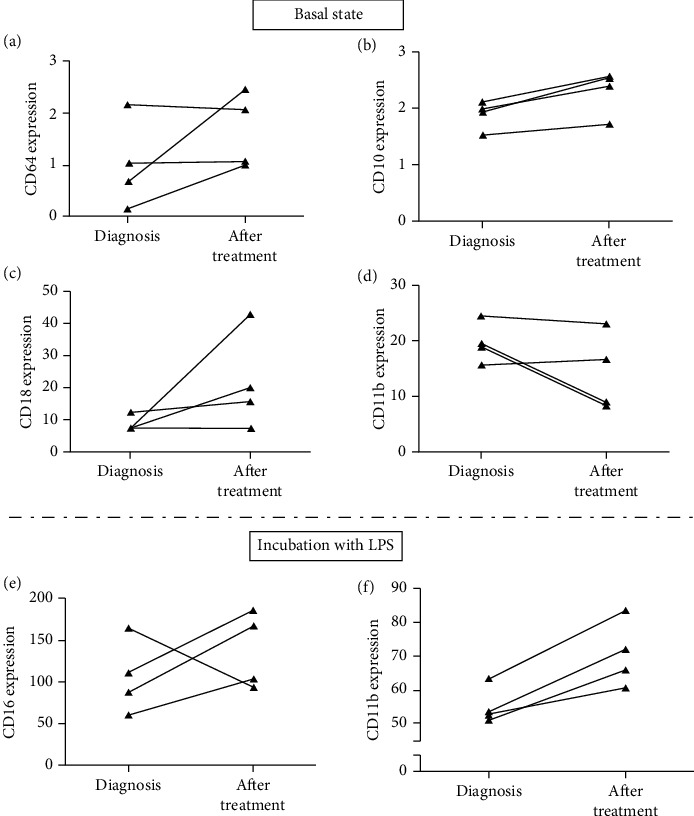
The intensity of expression of surface markers on PMN in four PAD patients before and after (5-7 months) the initiation of immunoglobulin replacement treatment. (a) CD64, (b) CD10, (c) CD18, and (d) CD11b at basal state and (e) CD16 and (f) CD11b after whole-blood incubation with LPS. Each line in the plot corresponds to data of each patient.

As mentioned above, four PAD patients at diagnosis were re-evaluated for their PMN immunophenotype, 5-7 months after the initiation of immunoglobulin replacement treatment. Interestingly, two out of four patients exhibited at basal state an upregulation of CD64 on PMN after treatment (Figure 3). Considering the other analyzed markers, a remarkable increase of CD10 expression on PMN was also observed in all patients at basal state; two out of four patients also displayed a decrease of CD11b and an increase of CD18 expression on PMN (Figure 3), while the other markers on PMN, including also CD16 on monocytes, did not follow a steady trend of expression (data not shown).

Moreover, these PAD patients after immunoglobulin replacement treatment displayed a remarkable increase of CD11b and CD16 expression on PMN after incubation with LPS (Figure 3). Consequently, the increased expression of CD11b found in our experiments in LPS-treated PAD patients' PMN might be indicative of a more sufficient activation capacity of PMN after immunoglobulin replacement treatment. Finally, in these newly diagnosed PAD patients, no significant correlations were found between the expression of Fc receptors (CD16 and CD64 on PMN and CD16 on monocytes) and their IgG levels after replacement treatment (*p* > 0.05, in all cases).

### 3.3. The Effect of Clinical Characteristics on PMN Immunophenotyping

Since PAD represent a heterogeneous group of disorders, we further evaluated whether specific clinical phenotypes affect the immunophenotype of innate immune cells. Interestingly, we identified that patients with splenomegaly exhibited a lower CD10 expression on PMN compared with those without splenomegaly (*p* = 0.030, Figure S2). Moreover, after PMN isolation, we observed that PAD patients with autoimmunity displayed a higher expression of CD11b, CD11c, and CD16 on PMN compared to patients without autoimmune manifestations (*p* = 0.001, *p* = 0.001, and *p* < 0.001, respectively; Figure S2). Finally, the expression markers on PMN and monocytes did not differ significantly in other clinical phenotypes of PAD patients in all experimental conditions (*p* > 0.05, in all cases).

### 3.4. Innate Immune Cell Immunophenotyping in a Patient during Different Clinical Conditions

**Figure 4:**
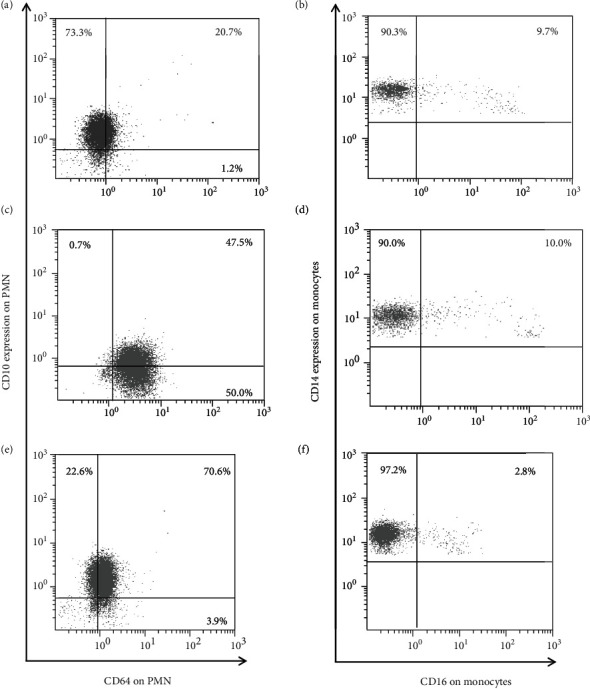
The intensity of expression of CD64 and CD10 on PMN (a, c, e) and CD14 and CD16 on monocytes (b, d, f), in a CVID patient in different clinical conditions. (a, b) At diagnosis. (c, d) Three months later during an upper respiratory infection with rash. (e, f) Rest state five months after the recovery of infection.

Among the patients analyzed, we had the opportunity to evaluate a newly diagnosed CVID patient, with severe hypogammaglobulinemia and an absence of antibody responses to vaccines, without any history of recurrent or chronic infections. The patient carried the *IKZF1-*p.His191Tyr mutation in heterozygous state (as mentioned in Subjects), and his diagnosis was established when his brother (family proband) was also diagnosed with CVID due to recurrent ATP and a history of recurrent skin infections. This patient refused immunoglobulin replacement treatment, and his whole-blood immunophenotyping was performed several times, in different clinical conditions, namely, (1) at diagnosis without infection, (2) during an upper respiratory infection with rash, and (3) at resting state without acute infection, eight months after the initial diagnosis. Interestingly enough, we demonstrated that the expression pattern of CD64 and CD10 on PMN differed substantially in different clinical conditions, while CD16 expression on monocytes remained low and stable (Figure 4). In particular, we observed that CD64 is upregulated and CD10 is downregulated during infection; however, while CD10 expression reverted to initial levels, the expression of CD64 on PMN remained upregulated five months after the resolution of acute infection (Figure 4). It is worth of note that although CD64 expression on PMN was significantly correlated with CD16 expression on monocytes in healthy controls, PAD patients did not follow the same pattern (Figure 2). While the majority of PAD patients displayed a simultaneous activation of PMN and monocytes, there are some patients, like him presented above, who displayed high expression of CD64 on PMN but low expression of CD16 on monocytes, or the opposite (Figure 2). These findings suggest a more profound role of innate immune cells immunophenotyping in PAD that is discussed in detail below.

## 4. Discussion

In this study, we clearly demonstrate that PAD patients display a persistent activation status of PMN and monocytes, indicative of a chronic inflammatory condition, while immunoglobulin replacement treatment does not modify substantially this activation status but seems to restore a more sufficient capacity of PMN to respond to LPS (and pathogen) exposure.

Our findings indicate that PMN of PAD patients display a more immature phenotype compared to healthy subjects. Additionally, they exhibit an activated status, although in a lower level compared to sepsis patients. In our study, we did consider the use of additional patient cohorts in order to evaluate the effect of immunoglobulin treatment, since the majority of them represent immune mediated conditions (i.e., patients with neurologic conditions, as multiple sclerosis). Although other systemic diseases affecting PMN physiology could also be considered patient control groups, we considered that the comparison of PAD patients to sepsis ones is more adequate, since sepsis has extensively been investigated in multiple studies, with reproducible data.

Our results are supported by the findings of some previous studies indicating an increased expression of activation markers, as elastase and myeloperoxidase (MPO) on PMN of CVID patients, especially in those displaying splenomegaly, while immunoglobulin treatment further induced PMN activation, in both in vivo and in vitro conditions [10]. In the same context, Vlkova et al. recently reported that CVID patients under immunoglobulin replacement treatment exhibited an increased gelatinase-associated lipocalin (NGAL) plasma concentration and immunophenotypic changes of PMN surface receptors, such as increased levels of CD11b and PD-L1, and decreased levels of CD62L, CD16, and CD80, which are also indicative of PMN steady activation status [11]. Interestingly, these findings are also supported by the results of the study of Casuli et al., suggesting that low concentrations of IVIG can induce PMN activation in healthy individuals [29].

Abnormalities observed in the innate immunity compartment might be associated PAD phenotype. PMN disturbances either inherent or epiphenomena may contribute to the increased susceptibility of PAD patients to infections, affecting directly the response to pathogens by attenuating functions such as phagocytosis, chemotaxis, and transmigration. On the other hand, these disturbances may also affect the dialectic communication of PMN with the other counterparts of the immune system, contributing also to the emergence and/or the perpetuation of the other inflammatory manifestations of the disease.

As mentioned above, the more impressive finding of our study was the upregulation of CD64 expression on PMN in our cohort of PAD patients, regardless of their treatment status. CD64 (Fc*γ*RI) is a high-affinity IgG receptor that is expressed only in activated granulocytes and is a useful marker indicative of infectious and septic conditions [30, 31]. Previous studies have shown that CD64 on PMN is induced by cytokines, such as G-CSF and IFN-gamma [32], while a high CD64 expression on PMN has been reported in patients with acute and chronic inflammatory conditions, such as bacterial infections, sepsis, and autoinflammatory disorders, including Familial Mediterranean Fever (FMF) and PFAPA (Periodic Fever, Aphthous Stomatitis, Pharyngitis, Adenitis) syndrome [30, 31, 33, 34]. On the other hand, CD64 is not highly upregulated on PMN in the setting of autoimmunity-related inflammation, as in patients with rheumatoid arthritis or lupus [33]. Considering PAD, there is only one previous study in the literature evaluating the expression of CD64 on PMN in CVID patients, suggesting that a high CD64 expression is associated with a worse prognosis and outcome [7].

On the other hand, there are several studies in the literature analyzing the expression of CD16 on PMN in CVID patients, with contradictory results [7–11]. CD16 (Fc*γ*RIII) is a low-affinity IgG receptor; there are two isoforms of CD16, the GPI-anchored CD16b which is expressed on PMN and its cross-linking results in their degranulation and the activation of respiratory burst, while CD16a is expressed on monocytes, macrophages, and NK cells [23, 24, 35]. Both isoforms of CD16 interact with CD11b/CD18 molecule-regulating phagocytosis, while CD16a has also been shown to mediate cytotoxicity by monocytes and NK cells [23, 24, 36]. Thon et al. have shown no significant differences of CD16 expression on PMN between CVID patients and healthy controls [7], while Casuli et al. described a lower CD16 expression on PMN in their cohort [8], similar with our findings described above. Moreover, Prezzo et al. did not observe any difference of CD16 expression on PMN before and after a course of immunoglobulin replacement treatment, suggesting that the replacement treatment does not affect PMN function [9].

An advantage of our study was the opportunity to analyze newly diagnosed patients before the initiation of replacement treatment and also several months later, and, obviously, our findings are more indicative of the effect of immunoglobulin treatment on PMN function. In this context, we also observed that several months after the initiation of immunoglobulin replacement treatment, PAD patients display an increase in the expression of CD11b integrin molecule as well as of CD16 on PMN, after LPS incubation, which is also indicative of their enhanced activation capacity after treatment. CD11b is an integrin molecule which along with CD18 represents a complement receptor, binding to C3bi and resulting in phagocytosis of opsonized pathogens. Moreover, CD11b along with CD66b resides in the specific PMN granules, and its expression represents a characteristic activation and degranulation marker [37]. However, due to the small number of patients analyzed, we consider that no safe conclusions can be drawn, and further studies, with a large cohort of PAD patients at diagnosis and after treatment, are necessary in order to clarify the effect of immunoglobulin replacement treatment on innate immune cell immunophenotyping.

As mentioned above, we observed a high CD16 expression on monocytes of PAD and sepsis patients, compared to healthy controls (Figure 1). CD16-positive monocytes represent the so-called proinflammatory subpopulation of intermediate and non-classical monocytes, which is observed enhanced in Gram-negative sepsis [38] and characterized by increased production of TNF-alpha and minimum levels of IL-10 [39]. Similar to our study, Barbosa et al. reported increased percentages of CD16-positive monocytes in CVID but not in X-linked agammaglobulinemia patients, suggesting that this finding might not be due to high levels of LPS, but rather to T and B cell aberrancies, which are common in patients with CVID [4].

In our study, we enrolled two patients carrying *CTLA4* mutations; these patients had an initial diagnosis of CVID, fulfilling the diagnostic criteria of the disease (low immunoglobulin levels, absence of antibody responses to vaccines, and exclusion of secondary causes of immunodeficiency), exhibiting also lymphoproliferation and autoimmune manifestations [21], and they received replacement treatment for years before the demonstration of *CTLA4* mutations. As it is well known, the molecular defects causing CVID are still unknown for the majority of patients, and the disease is rather a collection of different disorders with common clinical characteristics and laboratory findings [1, 15]. Over time, several patients with an initial diagnosis of CVID have finally been diagnosed with another disease entity, such as patients with *CTLA4*-induced immunodeficiency, who were initially considered suffering from CHAI syndrome [40], and nowadays, the disease is categorized within immune dysregulation syndromes [41]. However, as we observed for our patients with *CTLA4* mutations, their clinical and laboratory findings did not differ substantially compared to other CVID patients, and for this reason, we decided not to exclude them from our study. Furthermore, we did not observe any substantial differences considering PMN and monocyte immunophenotyping between *CTLA4*-mediated immunodeficiency and the rest of the PAD patients, in all experimental conditions analyzed.

The variable nature of PAD diseases is due to the distinct genetic background that cause and/or affect their clinical phenotype [1, 15]. This variability is also reflected in immunophenotypic characteristics of immune cells, as we also observed in our experiments. Indeed, PAD patients with splenomegaly exhibited lower CD10 expression on PMN suggesting a more immature phenotype; on the other hand, patients with autoimmune manifestations exhibited a more activated phenotype (displaying an increased integrin CD11b and CD16 expression on isolated PMN), suggesting an enhanced potential for arrest on the endothelium (and subsequent extravasation), as well as an enhanced potential for phagocytosis. In this context, Maggadottir et al. have recently reported that ITGAM (CD11b) gene polymorphisms are significantly associated with CVID, suggesting a more important role of CD11b in disease pathogenesis and/or phenotype [42]. On the other hand, there is increased evidence that the chronic activation of PMN aggravates tissue damage via ROS production [43]; the subsequent release of both toxic contents and inflammatory cytokines may result in a continuous exposure of self-epitopes and a predisposition to autoimmune manifestations.

In the end, we had the opportunity to evaluate a CVID untreated patient several times (Figure 4), and we demonstrated significant fluctuations in the expression of PMN markers, while the percentage of proinflammatory CD16-positive monocyte subset remained extremely low, even during inflammatory conditions. As mentioned above, this patient carried a *IKZF1* (Ikaros) mutation, and as it has been demonstrated by previous studies, CVID13 patients (carrying Ikaros mutations) show decreased percentages of CD16^+^ monocytes, as well as a defective dendritic cell maturation, affecting to a lesser degree the PMN maturation and function [44]. Consequently, we can deduce that certain phenotypic findings of immune cells in PAD patients might be associated with the primary genetic defect(s) that is (are) the cause(s) of the disease.

## 5. Conclusion

Our findings indicate that PAD patients exhibit a persistent activation of innate immune cells that might be the result of either intrinsic defects leading to primary immunodeficiency or/and the chronic inflammatory stress observed usually in these disorders. Moreover, the experimental manipulation of immune cells, along with the distinct clinical characteristics and the treatment status of PAD patients should always been taken into consideration, in order to draw safe conclusions for in vivo phenomena when extrapolating in vitro data.

## Data Availability

All data being analyzed in this manuscript are available upon request to the corresponding author.

## Supplementary Materials

Supplementary Materials Figure S1: CD64 intensity of expression on polymorphonuclear cells (PMN) in different experimental conditions in the patients and controls of the study: A. after PMN isolation, B. after whole-blood incubation without LPS, C. after whole-blood incubation with LPS of total PAD patients. The lines represent the median values. Statistical analyses were performed by the Mann–Whitney *U* test; ∗*p* < 0.05; ∗∗*p* < 0.01; ns: not significant. Figure S2: A. CD10 intensity of expression on polymorphonuclear cells (PMN) at basal state according to the presence of splenomegaly. B. CD11b, C. CD11c and D. CD16 intensity of expression on PMN after their isolation, according to the presence of autoimmunity. The lines represent the median values. Statistical analyses were performed by the Mann–Whitney *U* test; ∗*p* < 0.05; ∗∗*p* < 0.01; ns: not significant. Table S1: demographic, genetic, and clinical data of PAD patients of the study. Abbreviations: M, male; F, female; CRD, chronic respiratory disease; fSCIG, facilitated subcutaneous immunoglobulin; IVIG, intravenous immunoglobulin; SCIG, subcutaneous immunoglobulin. ^Patients 3-6 were also analyzed after the initiation of replacement treatment (see Materials and Methods for details). ∗Patient FL carried also the mutation *JAK3*-p.R840C (Ref. [21]). Table S2: monocyte counts and subpopulations in the patients and controls of the study. Abbreviations: HC, healthy controls, PAD, primary antibody deficiencies; PMN, polymorphonuclear cells; SDEV, standard deviation. Statistical analysis was performed by the Mann–Whitney *U* test, while *p* refers to comparison of PAD patients vs. HC, sepsis patients vs. HC, and PAD patients vs. sepsis patients. Table S3: expression of surface markers on PMN at basal state. Abbreviations: HC, healthy controls, PAD, primary antibody deficiencies; PMN, polymorphonuclear cells; SDEV, standard deviation. Statistical analysis was performed by the Mann–Whitney *U* test, while *p*1 refers to comparison of PAD patients vs. HC and *p*2 refers to the comparison of sepsis patients vs. HC. Table S4: expression of surface markers on PMN after isolation. Abbreviations: HC, healthy controls, PAD, primary antibody deficiencies; PMN, polymorphonuclear cells. Statistical analysis was performed by the Mann–Whitney *U* test, while *p* refers to comparison of PAD patients vs. HC. Table S5. Expression of surface markers on PMN after incubation without LPS. Abbreviations: HC, healthy donors, LPS, lipopolysaccharides; PAD, primary antibody deficiencies; PMN, polymorphonuclear cells. Statistical analysis was performed by the Mann–Whitney *U* test, while *p* refers to comparison of PAD patients vs. HC. Table S6. Expression of surface markers on PMN after incubation with LPS. Abbreviations: HC, healthy donors, LPS, lipopolysaccharides; PAD, primary antibody deficiencies; PMN, polymorphonuclear cells. Statistical analysis was performed by the Mann–Whitney *U* test, while *p* refers to comparison of PAD patients vs. HC.
