# Supplementary material for: Persistent Activation of Innate Immunity in Patients with Primary Antibody Deficiencies
Source: J Immunol Res. 2020 Nov 20;2020:8317671. doi: 10.1155/2020/8317671 (PMC7695510; doi:10.1155/2020/8317671)
Supplement: Supplementary Materials — Figure S1: CD64 intensity of expression on polymorphonuclear cells (PMN) in different experimental conditions in the patients and controls of the study: A. after PMN isolation, B. after whole-blood incubation without LPS, C. after whole-blood incubation with LPS of total PAD patients. The lines represent the median values. Statistical analyses were performed by the Mann–Whitney U test; ∗p < 0.05; ∗∗p < 0.01; ns: not significant. Figure S2: A. CD10 intensity of expression on polymorphonuclear cells (PMN) at basal state according to the presence of splenomegaly. B. CD11b, C. CD11c and D. CD16 intensity of expression on PMN after their isolation, according to the presence of autoimmunity. The lines represent the median values. Statistical analyses were performed by the Mann–Whitney U test; ∗p < 0.05; ∗∗p < 0.01; ns: not significant. Table S1: demographic, genetic, and clinical data of PAD patients of the study. Abbreviations: M, male; F, female; CRD, chronic respiratory disease; fSCIG, facilitated subcutaneous immunoglobulin; IVIG, intravenous immunoglobulin; SCIG, subcutaneous immunoglobulin. ^Patients 3-6 were also analyzed after the initiation of replacement treatment (see Materials and Methods for details). ∗Patient FL carried also the mutation JAK3-p.R840C (Ref. [21]). Table S2: monocyte counts and subpopulations in the patients and controls of the study. Abbreviations: HC, healthy controls, PAD, primary antibody deficiencies; PMN, polymorphonuclear cells; SDEV, standard deviation. Statistical analysis was performed by the Mann–Whitney U test, while p refers to comparison of PAD patients vs. HC, sepsis patients vs. HC, and PAD patients vs. sepsis patients. Table S3: expression of surface markers on PMN at basal state. Abbreviations: HC, healthy controls, PAD, primary antibody deficiencies; PMN, polymorphonuclear cells; SDEV, standard deviation. Statistical analysis was performed by the Mann–Whitney U test, while p1 refers to comparison of PAD patients vs. HC and p [file 8317671.f1.pdf]

Supplementary Material

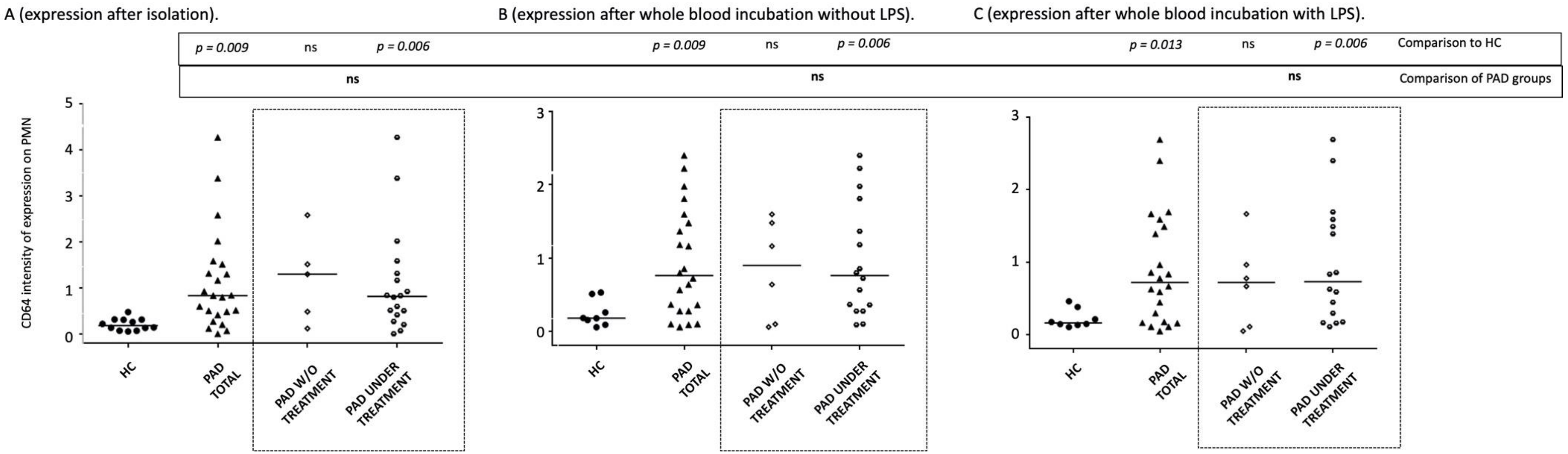

Supplementary Figure 1. CD64 intensity of expression on polymorphonuclear cells (PMN) in different experimental conditions in the patients and controls of the study: A. after PMN isolation, B. after whole blood incubation without LPS, C. after whole blood incubation with LPS of total PAD patients. The lines represent the median values. Statistical analyses were performed by Mann-Whitney U test; ∗ : when $p < 0.05$; ∗∗ : when $p < 0.01$; ns: not significant

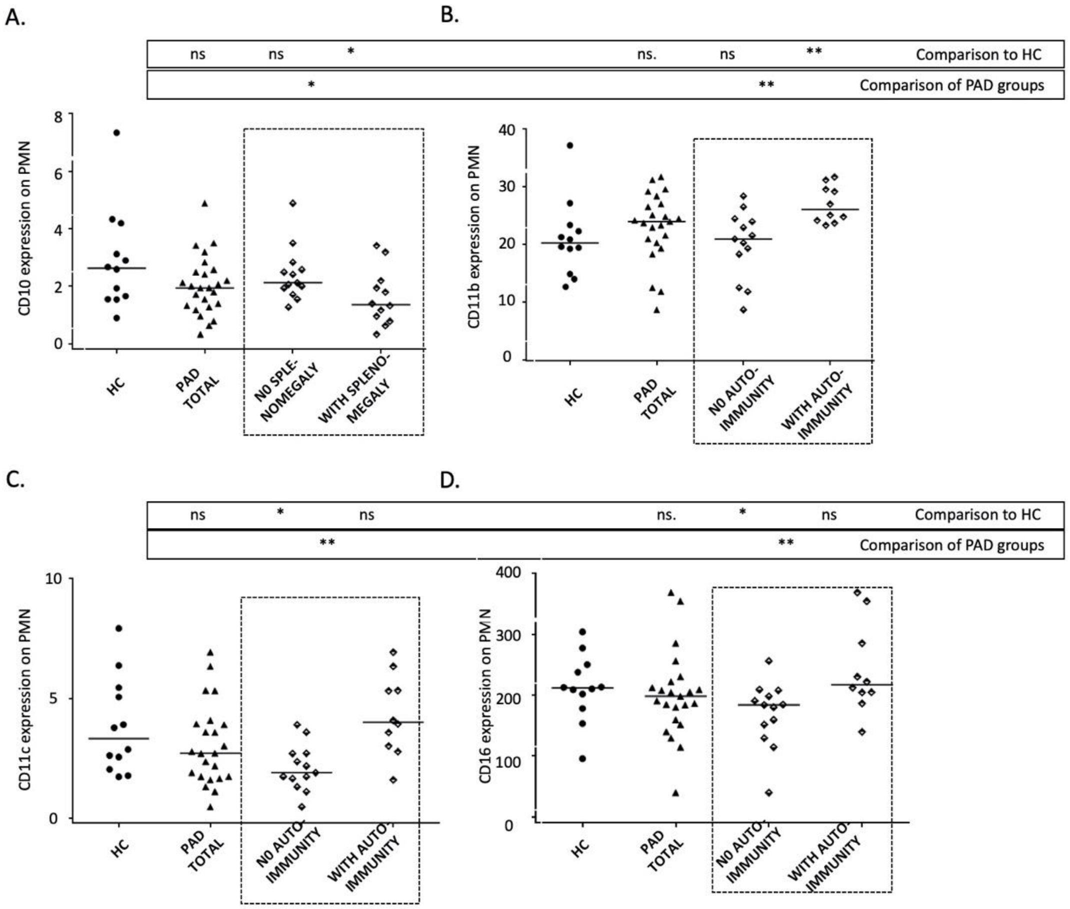

Supplementary Figure 2. A. CD10 intensity of expression on polymorphonuclear cells (PMN) at basal state according to the presence of splenomegaly. B. CD11b, C. CD11c and D. CD16 intensity of expression on PMN after their isolation, according to the presence of autoimmunity. The lines represent the median values. Statistical analyses were performed by Mann-Whitney U test; ∗ : when $p < 0.05$; ∗∗ : when $p < 0.01$; ns: not significant

Supplementary Table 1. Demographic, genetic and clinical data of PAD patients of the study

No	Patient	Treatment status	Sex	Age	Mutational status	Autoimmunity	CRD	Bronchiectasis	Enteropathy	Granulomas	Neoplasia	Spleenomegaly
1	D.K.	NO treatment	M	14	***IKZF1*-p.H191Y**	no	no	no	no	no	no	no
2	H.A.	NO treatment	M	48	Unknown	no	yes	yes	no	no	no	no
3	K.M.^	NO treatment	F	53	Unknown	no	no	no	yes	no	no	no
4	M.M.^	NO treatment	F	43	Unknown	yes	yes	yes	no	no	no	splenectomy
5	S.M.^	NO treatment	F	66	Unknown	no	no	yes	yes	no	no	no
6	T.D.^	NO treatment	F	43	Unknown	yes	no	no	no	no	yes	yes
7	A.A.	fSCIG	M	48	Unknown	no	yes	yes	no	no	no	no
8	D.M.	fSCIG	F	38	Unknown	no	no	no	no	yes	no	yes
9	T.H.	IVIG	F	40	Unknown	no	no	yes	no	yes	no	yes
10	K.A.	fSCIG	F	34	Unknown	no	no	no	yes	no	no	yes
11	K.M.	fSCIG	F	36	Unknown	yes	no	no	yes	no	no	no
12	K.B.	fSCIG	F	56	Unknown	no	yes	yes	no	no	no	no
13	K.Z.	fSCIG	F	65	Unknown	no	yes	no	no	yes	no	no
14	K.M.	fSCIG	F	39	Unknown	no	no	yes	no	no	no	yes
15	K.M.	IVIG	M	51	***TACI*-p.C104R**	yes	no	yes	yes	yes	yes	splenectomy
16	K.S.	fSCIG	M	28	Unknown	yes	yes	no	no	no	no	yes
17	M.D.	fSCIG	F	19	***CTLA4*-p.Y89X**	yes	no	no	yes	no	no	no
18	B.K.	fSCIG	F	49	Unknown	yes	no	yes	no	no	no	no
19	N.B.	IVIG	F	58	Unknown	no	no	no	no	no	no	yes
20	N.E.	IVIG	F	42	Unknown	no	yes	yes	no	no	no	yes
21	S.N.	fSCIG	M	30	***TACI*-p.C104R**	yes	yes	yes	no	no	no	yes
22	F.I.*	fSCIG	M	23	***CTLA4*-p.Y139C**	yes	no	no	no	no	no	splenectomy
23	M.E.	fSCIG (low CS dosage)	F	38	Unknown	no	yes	yes	no	no	no	yes
24	P.A.	fSCIG (and mycophenolate)	M	21	Unknown	yes	no	no	no	yes	no	yes
25	M.D.	fSCIG	M	51	***TACI*-p.C104R**	yes	no	no	no	no	no	yes

Abbreviations: M, male; F, female; CRD, chronic respiratory disease; fSCIG, facilitated subcutaneous immunoglobulin; IVIG, intravenous immunoglobulin; SCIG, subcutaneous immunoglobulin

^ Patients 3-6 were also analyzed after the initiation of replacement treatment (see Materials and Methods section for details)

* Patient FL carried also the mutation *JAK3*-p.R840C (Ref. 21, Frontiers in Immunology 2017;8:1824)

Supplementary Table 2. Monocyte counts and subpopulations in the patients and controls of the study

**A. MONOCYTES SUBPOPULATIONS (%)**				**HC vs PAD**	**HC vs SEPSIS**	**PAD VS SEPSIS**
	**HC** (n=12)	**PAD** (n=25)	**SEPSIS** (n=4)	**p**	**p**	**p**
**Classical** (mean, SDEV)	78.1 ± 17.3	65.6 ± 16.0	62.9 ± 16.5	**0.018**	0.133	0.818
**Intermediate** (mean,SDEV)	14.6 ± 14.1	24.7 ± 14.6	29.6 ± 17.0	**0.018**	0.103	0.584
**Non clasical** (mean, SDEV)	6.1 ± 4.0	8.1 ± 3.8	6.8 ± 4.0	0.106	0.684	0.671

**B. MONOCYTES SUBPOPULATIONS ($x10^9$/L of Whole Blood)**				**HC vs PAD**	**HC vs SEPSIS**	**PAD VS SEPSIS**
	**HC**	**PAD**	**SEPSIS**	**p**	**p**	**p**
**Monocytes total count** (mean, SDEV)	0.305 ± 0.123	0.375 ± 0.245	0.325 ± 0.206	0.772	0.932	0.825
**Classical** (mean, SDEV)	0.247 ± 0.105	0.263 ± 0.215	0.179 ± 0.083	0.402	0.262	0.865
**Intermediate** (mean, SDEV)	0.035 ± 0.025	0.080 ± 0.050	0.120 ± 0.109	**0.010**	0.262	0.542
**Non clasical** (mean, SDEV)	0.018 ± 0.013	0.026 ± 0.016	0.021 ± 0.023	0.185	>0.999	0.382

Abbreviations: HC, healthy controls, PAD, primary antibody deficiencies; PMN, polymorphonuclear cells; SDEV, standard deviation. Statistical analysis was performed by Mann-Whitney U-test. Statistical analysis was performed by Mann-Whitney U-test, while *p* refers to comparison of PAD patients vs HC, Sepsis patients vs HC and PAD patients vs Sepsis patients.

Supplementary Table 3. Expression of surface markers on PMN at basal state

Markers	HC (n 12)	Patients with sepsis (n 4)	PAD patients (n 25)	*p1*	*p2*
Absolute number ($x10^9/L$) (mean, SDEV)	4.5 ± 1.8	9.3 ± 2.8	4.5 ± 2.5	0.586	**0.011**
CD16 (mean, SDEV)	175.6 ± 41.7	81.6 ± 32.0	153.7 ± 43.0	0.052	**0.004**
CD18 (mean, SDEV)	9.2 ± 4.3	10.9 ± 4.9	9.7 ± 4.3	0.747	0.684
CD11b (mean, SDEV)	15.6 ± 7.0	14.9 ± 3.9	16.4 ± 4.9	0.336	0.951
CD66b (mean, SDEV)	5.6 ± 1.5	12.6 ± 5.9	6.2 ± 2.1	0.387	**0.002**
CD64 (mean, SDEV)	0.2 ± 0.1	1.8 ± 1.3	1.0 ± 0.7	**< 0.001**	**0.001**
CD10 (mean, SDEV)	2.9 ± 1.8	0.9 ± 0.9	2.0 ± 1.0	0.160	**0.019**
CD11c (mean, SDEV)	2.2 ± 1.2	1.9 ± 1.0	1.7 ± 1.0	0.195	0.782

Abbreviations: HC, healthy controls, PAD, primary antibody deficiencies; PMN, polymorphonuclear cells; SDEV, standard deviation. Statistical analysis was performed by Mann-Whitney U-test, while *p1* refers to comparison of PAD patients vs HC and *p2* refers to the comparison of sepsis patients vs HC.

Supplementary Table 4. Expression of surface markers on PMN after isolation

Markers	HC (n 12)	PAD patients (n 23)	*p*
CD16 (mean, SDEV)	212.1 ± 54.9	200.7 ± 71.1	0.293
CD18 (mean, SDEV)	14.4 ± 7.7	16.2 ± 7.6	0.493
CD11b (mean, SDEV)	21.0 ± 6.5	23.0 ± 6.0	0.139
CD66b (mean, SDEV)	9.1 ± 2.8	8.5 ± 4.1	0.716
CD64 (mean, SDEV)	0.2 ± 0.1	1.1 ± 1.1	**< 0.001**
CD10 (mean, SDEV)	5.7 ± 2.4	4.6 ± 2.3	0.151
CD11c (mean, SDEV)	3.8 ± 2.0	3.1 ± 1.7	0.262

Abbreviations: HC, healthy controls, PAD, primary antibody deficiencies; PMN, polymorphonuclear cells. Statistical analysis was performed by Mann-Whitney U-test, while *p* refers to comparison of PAD patients vs HC.

Supplementary Table 5. Expression of surface markers on PMN after incubation without LPS

Markers	HC<br>(n 12)	PAD patients<br>(n 25)	*p*
CD16 (mean, SDEV)	214.2 ± 38.5	174.6 ± 61.0	**0.012**
CD18 (mean, SDEV)	12.5 ± 7.1	12.5 ± 6.1	0.736
CD11b (mean, SDEV)	35.3 ± 5.8	36.1 ± 10.1	0.630
CD66b (mean, SDEV)	11.1 ± 3.3	12.0 ± 6.5	0.783
CD64 (mean, SDEV)	0.2 ± 0.2	0.9 + 0.7	**0.009**
CD10 (mean, SDEV)	4.4 ± 1.5	3.6 ± 1.8	0.393

Abbreviations: HC, healthy donors, LPS, lipopolysaccharides; PAD, primary antibody deficiencies; PMN, polymorphonuclear cells. Statistical analysis was performed by Mann-Whitney U-test, while *p* refers to comparison of PAD patients vs HC.

Supplementary Table 6. Expression of surface markers on PMN after incubation with LPS

Markers	HC<br>(n 12)	PAD patients<br>(n 25)	*p*
CD16 (mean, SDEV)	96.3 ± 34.8	102.5 ± 44.4	0.723
CD18 (mean, SDEV)	14.2 ± 6.8	13.5 ± 5.8	0.266
CD11b (mean, SDEV)	60.0 ± 5.7	59.1 ± 15.3	0.630
CD66b (mean, SDEV)	39.3 ± 18.4	34.3 ± 18.0	0.431
CD64 (mean, SDEV)	0.2 ± 0.1	0.9 + 0.8	**0.013**
CD10 (mean, SDEV)	4.3 ± 1.1	3.3 ± 1.7	0.107

Abbreviations: HC, healthy donors, LPS, lipopolysaccharides; PAD, primary antibody deficiencies; PMN, polymorphonuclear cells. Statistical analysis was performed by Mann-Whitney U-test, while *p* refers to comparison of PAD patients vs HC.
